# Supplementary material for: Evaluation of at-home methods for N95 filtering facepiece respirator decontamination
Source: Sci Rep. 2021 Oct 5;11:19750. doi: 10.1038/s41598-021-99129-8 (PMC8492771; doi:10.1038/s41598-021-99129-8)
Supplement: Supplementary file 1 — Supplementary Information. [file 41598_2021_99129_MOESM1_ESM.pdf]

Title:

Evaluation of at-home methods for N95 filtering facepiece respirator decontamination

One sentence summary:

Survey of at-home methods for N95 respirator decontamination using heat and evaluation of their effects on N95 respirator integrity.

Authors:

Chen, T.X. [1] *, Pinharanda, A. [2] *, Steinemann, N. A. [1], Yasuma-Mitobe, K. [3], Lee, E. [4], Hahn J. [5], Wu L. [4], Fanourakis, S. [6], Peterka, D. S. [1] †, Hillman, E.M.C [1,5,7] †

* These authors contributed equally to this work

† Corresponding authors. Emails: eh2245@columbia.edu, dp2403@columbia.edu

Affiliations:

[1] Mortimer B. Zuckerman Mind Brain Behavior Institute, New York, NY, 10027, USA

[2] Department of Biological Sciences, Columbia University, New York, NY 10027, USA

[3] Department of Microbiology and Immunology, Columbia University, New York, NY 10032, USA

[4] Columbia College, Columbia University, New York, NY 10032, USA

[5] Department of Biomedical Engineering, Columbia University, New York, NY 10027, USA

[6] Environmental Health and Safety, Columbia University, New York, NY 10032, USA

[7] Department of Radiology, Columbia University, New York, NY 10027, USA

LIST OF SUPPLEMENTARY MATERIALS:

- Supplementary Figs S1 – S5
- Supplementary Tables S1 – S10

SUPPLEMENTARY FIGURES:

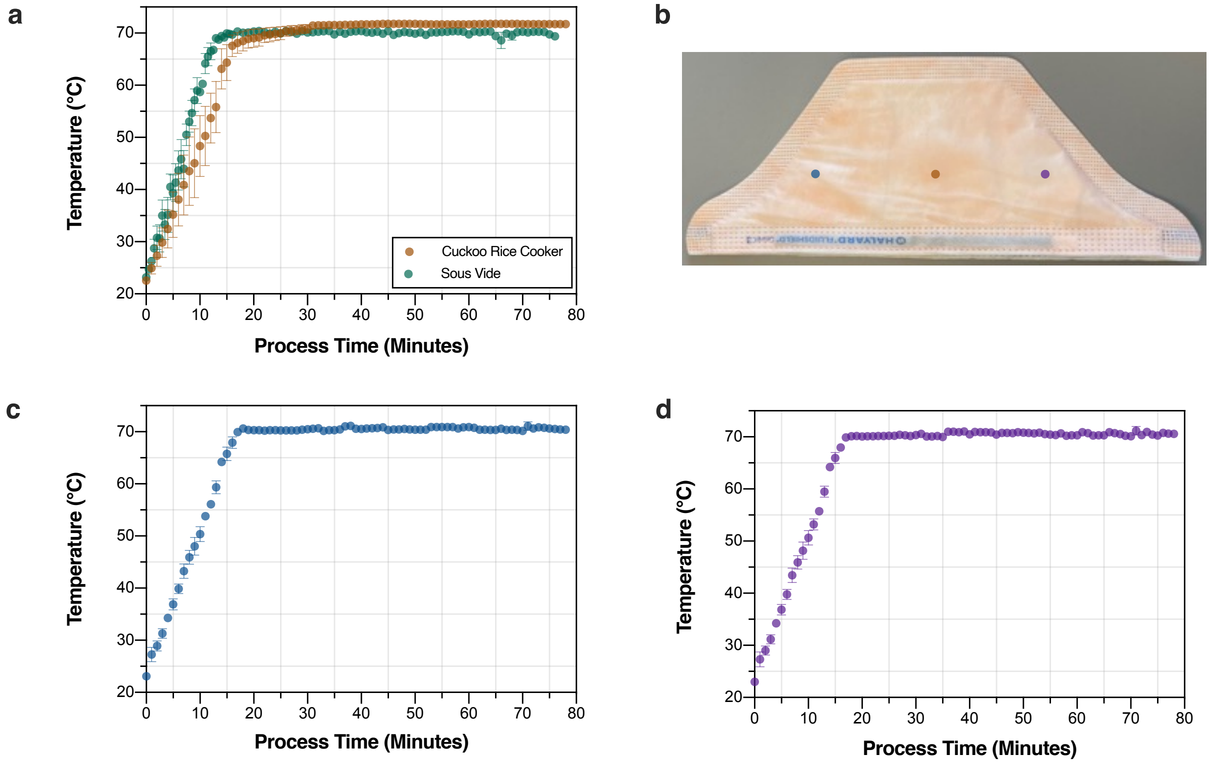

Fig S1. Average temperature profile of FFRs over one round of treatment. (a) Temperature was recorded for the duration of the treatment using a wireless sensor (Inkbird Thermometer IBS-TH1 for the rice cooker and MeatStick Wireless Thermometer 4335995327 for the sous vide machine). **(b)** The temperature was measured at two lateral locations of a control mask (blue and purple dots) in addition to the medial location (orange dot) previously used in **(a)**. **(c** and **d)** Measurements at each lateral position was recorded for the duration of one round of treatment and the average temperature profiles over three replicate measurements are shown.

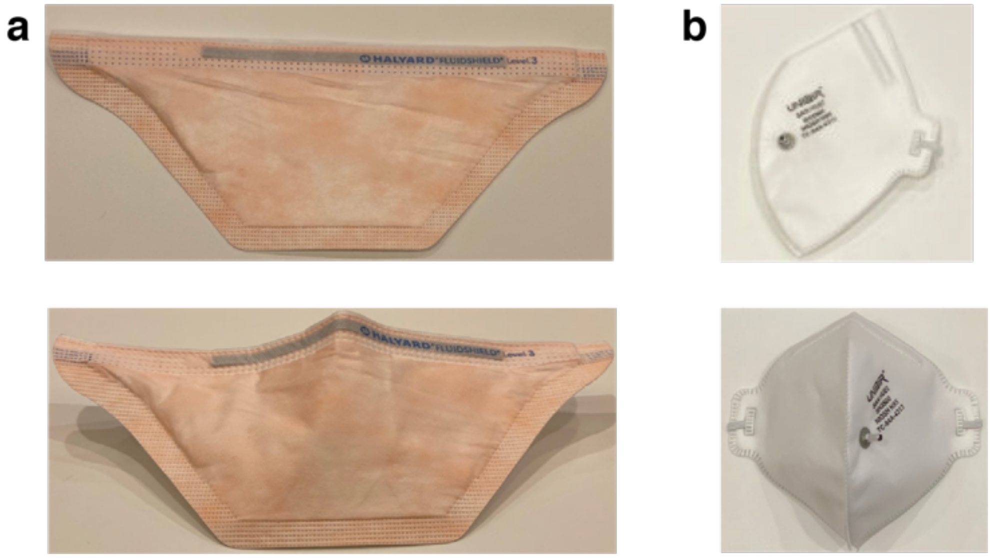

Fig S2. FFR models. (a) Halyard Fluidshield 3 FFR. **(b)** Uniair San Huei FFR. Port for quantitative fit testing visible in the photo.

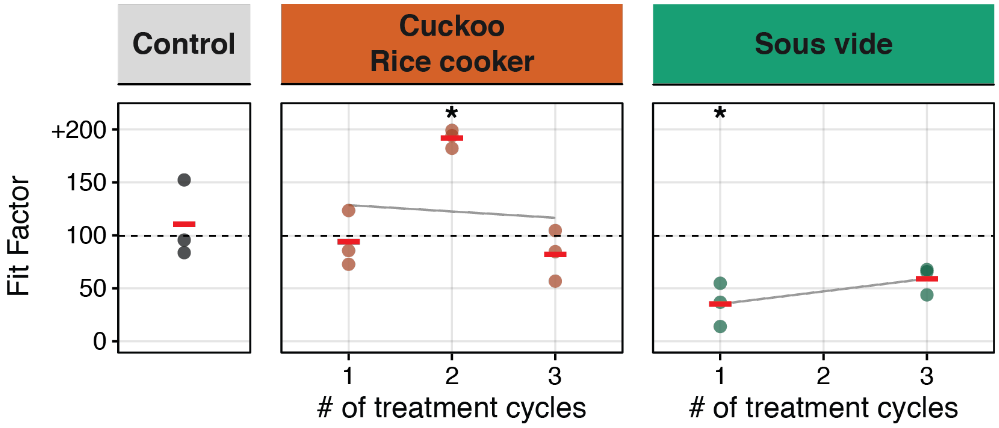

Fig S3. Average Uniair San Huei FFR fit factor before and after treatment. Overall fit factor as a function of treatment cycles for each condition: control (left), using a rice cooker (center) and a sous vide machine (right). Fit factor measures were repeated three times for each FFR (individual dots) and average fit factor is reported to the nearest integer (red bar). Grey regression lines quantify fit factor as a function of the number of treatment cycles (Table S4). Asterisks indicate significant differences in fit between treatment group and control (ANOVA, $p < 0.05$).

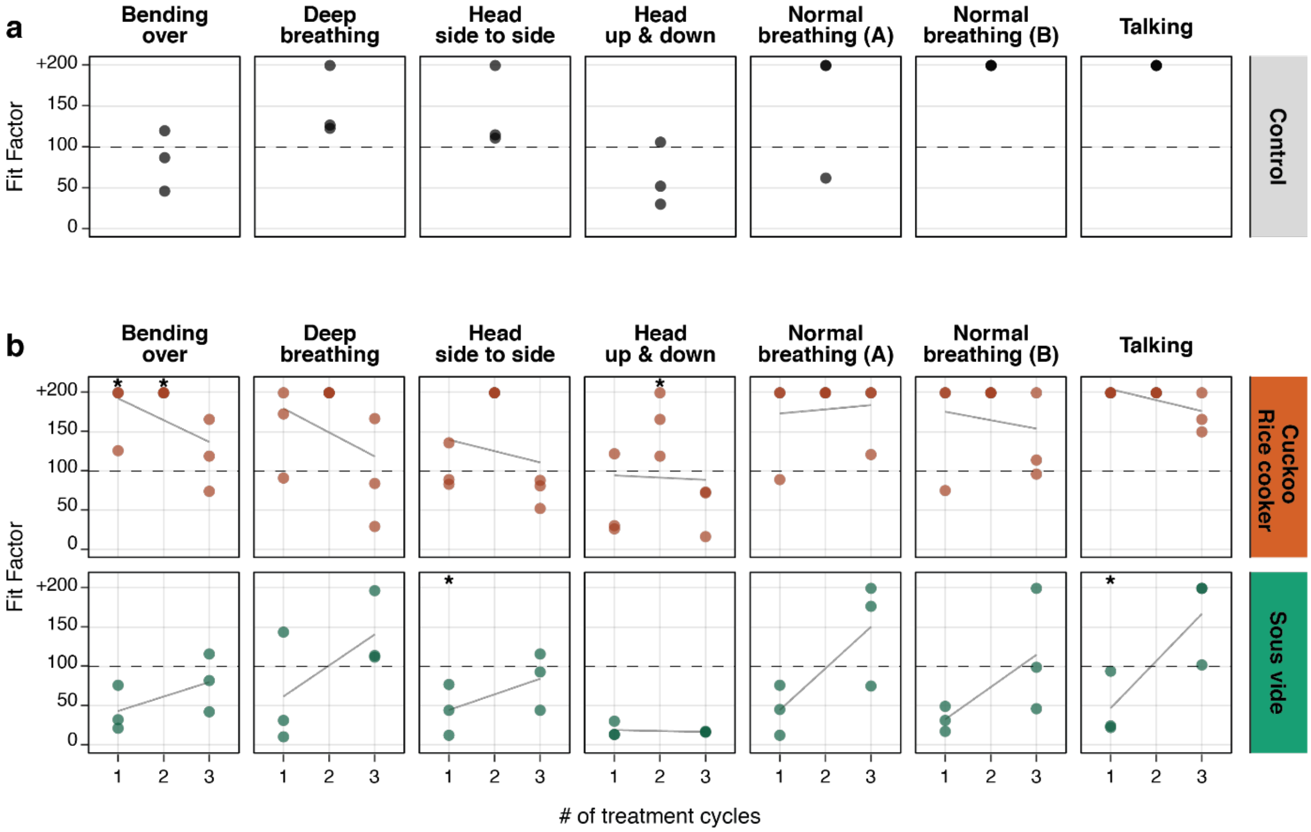

Fig S4. Uniair San Huei FFR fit for seven standardized testing conditions. The fit of control **(a)** and heat-treated **(b)** FFRs was assessed three times in each of seven testing conditions (individual dots) for each heat-treatment and control group. Grey lines in (b) signify linear regression fit of fit factor over treatment cycle (Table S5). Asterisks indicate significant differences in mean fit between treatment group and control (ANOVA, $p < 0.05$).

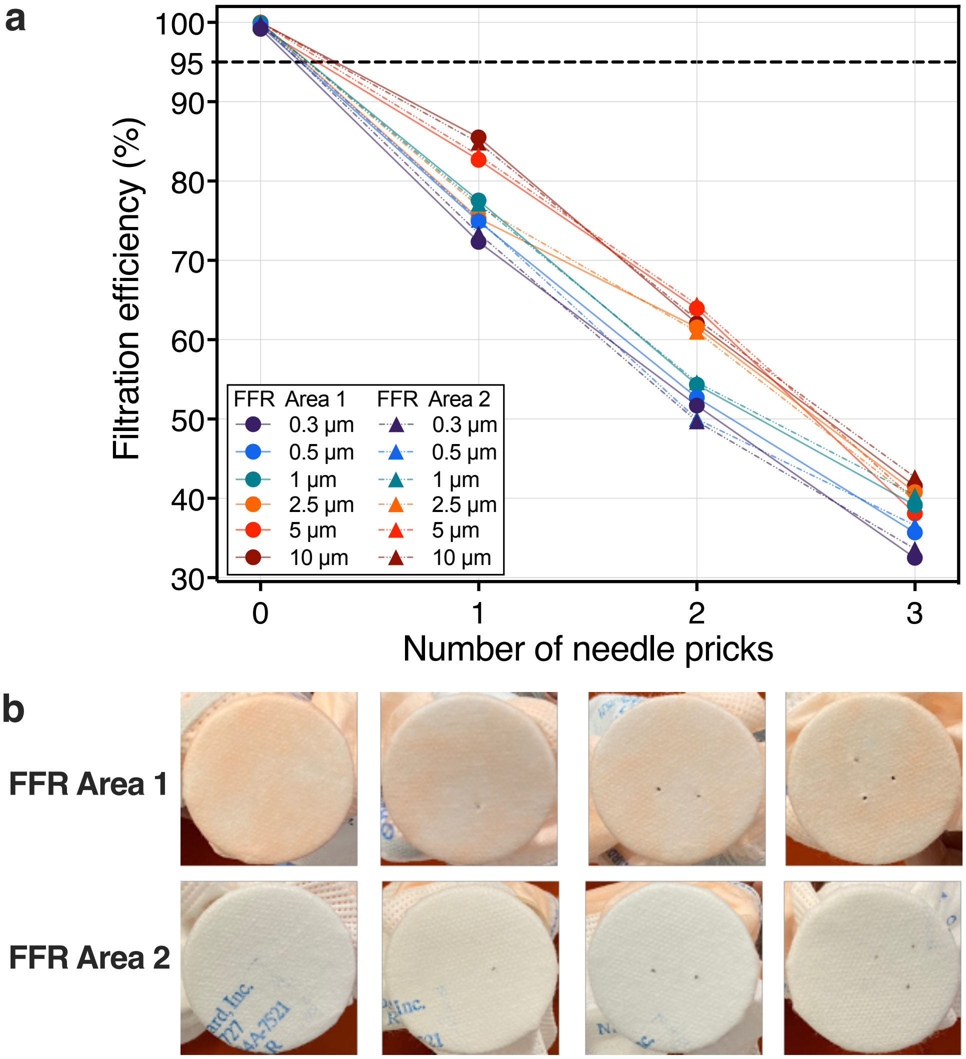

Fig S5. Filtration efficiency for control and punctured Halyard Fluidshield 3 FFRs. (a) The filtration efficiency of two N95 FFR areas for different particle sizes (bins of 0.3 µm, 0.5 µm, 1 µm, 2.5 µm, 5 µm, and 10 µm size classes) were determined for an untreated FFR before and after puncturing with an 18-gauge needle once, twice, and three times. Any number of needle pricks decreased the filtration efficiency below the 95% requirement. **(b)** The front view of two FFR regions subject to punctures, fitted to the sample holder, and tested for filtration efficiency is shown.

Supplementary Tables S1 – S10

Table S1. Effect of heat and humidity on enveloped viruses on N95 FFRs. Log viral reduction >3 is sufficient to consider the virus inactivated. N/A: Not Applicable, the parameter was not tested. DMEM refers to Dulbecco's Modified Eagle Medium.

Virus	Medium	Model	Method	Temp (ºC)	Humidity (%RH)	Time (min)	log reduction in virus	Filtration test passed after # treatments	Fit test passed after # treatments	Source
SARS-CoV-2	N/A	3M 1860S, 8110S, 8210S, 9105S	Dry heat	70	50	60	> 3	10	5	1
		AO safety N9504C				30	Insufficient	N/A	N/A	
						60	> 3.3	N/A	N/A	
	Bovine serum albumin, tryptone, mucin	3M 1860, 3M 1870, 3M Vflex 1804, AO Safety 1054	Steam	121	N/A	15	> 4.6-5.6	1-10	4	2
	DMEM	AOSafety N9504C	Dry heat	70	N/A	60	> 3	2	N/A	3
Murine coronavirus MHV	DMEM	3M 1860	Dry heat	72	1	30	Insufficient	N/A	N/A	4
				82	25					
				72	25		> 3.5			
		3M 8210	Humid heat	75	90	30	> 6	N/A	10	5
H5N1	Aerosolized allantonic fluid	3M 1860S, 3M 1870	Humid heat	65	N/A	30	> 4.62-4.65	N/A	N/A	6

Table S2. Temperature maintained after initial heating period in the appliances tested. The Cuckoo (CR-0655F) *Keep Warm* setting maintained an average temperature of 71.7°C (-2.0°C /+0.5°C). The sous vide AuAg Immersion Circulator (A808, 950 W) has an average temperature of 69.8°C (-1.6°C /+0.4°C). The Aroma (ARC-743-1NGB) *Keep Warm* setting reached an average temperature of 67.5°C (-4.0°C /+2.0°C).

**Type**	**Appliance**	**Temperature (°C)**		
		**Min**	**Mean**	**Max**
Rice cooker	Cuckoo (CR-0655F)	69.7	71.7	72.2
	Aroma (ARC-743-1NGB)	63.5	67.5	69.5
Sous vide	AuAg Immersion Circulator (A808, 950 W)	68.5	70.1	70.5

Table S3. Average Halyard Fluidshield 3 FFR fit factor and technical replicates. After exposing FFRs to several methods and rounds of treatment, the fit factor was estimated with the PortaCountPro+. The fit factor was estimated three times for each FFRs and the average fit factor is reported to the nearest integer. Control FFR was not subjected to any treatment. Analysis of variance p-values shown for variation in means among treatment group and control. Values rounded to two decimal places. Grey regression line overlaid for each treatment method and category (y ~ mx + b) in Fig 2.

Rounds of treatment	Treatment	Fit Factor			Average Fit Factor	p-value	F statistic	Regression line and r^2
		Technical replicate 1	Technical replicate 2	Technical replicate 3				
0	Control	108	184	94	129	-	-	-
1	Boil	51	141	105	99	0.02	12.54	$y = 131 - 31x$, $r^2 = 0.99$
2		54	86	73	71	0.00	123.67	
3		33	55	23	37	0.00	196.67	
1	Cuckoo Rice cooker	+200	167	175	181	0.31	1.32	$y = 187 - 4.1x$, $r^2 = 0.67$
2		167	192	187	182	0.28	1.53	
3		165	+200	154	173	0.24	1.93	
4		184	173	176	178	0.08	5.70	
5		144	143	+200	162	0.18	2.57	
1	Sous vide	129	+200	188	172	0.39	0.91	$y = 206 - 22.8x$, $r^2 = 0.66$
2		49	186	199	145	0.37	1.04	
3		+200	+200	137	179	0.53	0.47	
4		78	98	193	123	0.12	3.89	
5		72	76	59	69	0.00	36.76	

Table S4. Average FFR fit factor and technical replicates Uniair San Huei
After exposing FFRs to several methods and rounds of treatment, the fit factor was estimated with the PortaCountPro+. The fit factor was estimated three times for each FFRs and the average fit factor is reported to the nearest integer. The control FFR was not subjected to any round of treatment. Analysis of variance p-values shown for variation in means among treatment group and control. Values rounded to two decimal places. Grey regression line overlaid for each treatment method and category (y ~ mx + b) in Fig S3.

Rounds of treatment	Treatment	Fit Factor			Average Fit Factor	p-value	F statistic	Regression line and r^2 (x=reps)
		Technical replicate 1	Technical replicate 2	Technical replicate 3				
0	Control	96	84	153	111	-	-	-
1	Cuckoo Rice cooker	73	86	124	94.3	0.56	0.4	y = 135–6x, r^2 = 0.01
2		183	+200	195	192.6	0.02	13.94	
3		85	105	57	82.3	0.32	1.27	
1	Sous vide	55	37	14	35.3	0.04	9.64	y = 23.3–12x
2		-	-	-	-	-	-	
3		73	86	124	59.3	0.08	5.21	

Table S5. Analysis of variance among treatment category groups and control Uniair San Huei. F- and p-values denote the variation in means among treatment category groups and control in an analysis of variance. Grey regression line overlaid for each treatment method and category (y ~ mx + b) in Fig S4.

Treatment	Category	Rounds of treatment	p-value	F statistics	Regression line and r^2, (x=reps)
Cuckoo Rice cooker	Bending over	1	0.05	7.76	$y = 221 - 28.8x$, $r^2 = 0.26$
		2	0.01	29.20	
		3	0.36	1.07	
	Deep breathing	1	0.92	0.01	$y = 211 - 30.7x$, $r^2 = 0.17$
		2	0.12	3.99	
		3	0.30	1.44	
	Head side to side	1	0.31	1.38	$y = 154 - 14.5x$, $r^2 = 0.04$
		2	0.12	3.99	
		3	0.09	4.84	
	Head up & down	1	0.94	0.01	$y = 97.2 - 2.83x$, $r^2 = 0.001$
		2	0.04	9.23	
		3	0.77	0.09	
	Normal breathing (A)	1	0.89	0.02	$y = 168 + 5.33x$, $r^2 = 0.01$
		2	0.37	1.00	
		3	0.73	0.14	
	Normal breathing (B)	1	0.37	1.00	$y = 187 - 10.8x$, $r^2 = 0.03$
		2	0.37	1.00	
		3	0.12	3.89	
	Talking	1	0.37	1.00	$y = 219 - 14x$, $r^2 = 0.41$
		2	0.37	1.00	
		3	0.13	3.61	
Sous vide	Bending over	1	0.20	2.31	$y = 24.5 + 18.5x$, $r^2 = 0.32$
		3	0.89	0.02	
	Deep breathing	1	0.14	3.31	$y = 22 + 39.7x$, $r^2 = 0.39$
		3	0.82	0.06	
	Head side to side	1	0.05	7.98	$y = 24.3 - 20x$, $r^2 = 0.33$
		3	0.18	2.57	
	Head up & down	1	0.13	3.57	$y = 19.8 - 1.17x$, $r^2 = 0.04$
		3	0.11	4.21	
	Normal breathing (A)	1	0.09	4.89	$y = -8.83 + 53.2x$, $r^2 = 0.61$
		3	0.96	0.00	
	Normal breathing (B)	1	0.00	327.65	$y = -9 + 41.3x$, $r^2 = 0.45$
		3	0.13	3.54	
	Talking	1	0.00	41.95	$y = -13.7 + 60.3x$, $r^2 = 0.69$
		3	0.37	1.00	

Table S6. Halyard Fluidshield 3 FFR fit for different testing categories. After exposing FFRs to several methods and rounds of treatment, the fit for different testing categories was estimated with the PortaCountPro+. The fit was estimated three times for each category. Control FFRs was not subjected to any round of treatment.

Rounds of treatment	Treatment	Technical replicate	Bending over	Deep breathing	Head side to side	Head up & down	Normal breathing (A)	Normal breathing (B)	Talking
0	Control	1	52	116	111	126	178	123	181
		2	122	+200	+200	+200	+200	+200	+200
		3	57	127	119	81	112	111	99
1	Boil	1	63	28	42	98	42	87	63
		2	127	118	128	146	163	172	154
		3	99	108	96	120	79	124	128
2		1	51	52	53	48	71	88	42
		2	73	116	63	65	180	110	78
		3	48	55	72	84	49	+200	178
3		1	61	20	32	39	26	72	26
		2	51	64	69	47	55	52	52
		3	27	13	30	18	46	20	35
1	Cuckoo Rice cooker	1	+200	+200	+200	+200	+200	+200	+200
		2	+200	+200	83	+200	+200	+200	+200
		3	150	119	+200	+200	+200	+200	+200
2		1	+200	+200	+200	+200	133	+200	+200
		2	+200	+200	+200	+200	+200	+200	154
		3	+200	+200	+200	159	+200	95	+200
3		1	168	167	+200	+200	101	183	+200
		2	+200	+200	+200	+200	+200	+200	+200
		3	65	+200	+200	+200	+200	+200	+200
4		1	133	+200	+200	+200	+200	177	+200
		2	124	185	148	185	+200	+200	+200
		3	195	+200	+200	+200	133	138	+200
5		1	74	+200	183	+200	+200	121	162
		2	100	136	+200	140	109	+200	+200
		3	+200	+200	+200	+200	+200	+200	+200
1	Sous vide	1	188	153	+200	44	+200	+200	+200
		2	+200	+200	+200	+200	+200	+200	+200
		3	148	+200	181	+200	+200	+200	+200
2		1	27	56	47	56	68	59	63
		2	136	+200	+200	+200	190	+200	+200

		3	190	+200	+200	+200	+200	+200	+200
3		1	+200	+200	+200	+200	198	+200	+200
		2	+200	+200	+200	+200	+200	+200	+200
		3	+200	+200	78	+200	+200	75	+200
4		1	32	110	99	72	63	46	114
		2	76	116	99	92	108	99	110
		3	159	+200	+200	+200	+200	197	+200
5		1	68	45	77	123	99	199	43
		2	37	135	56	77	133	110	108
		3	57	62	51	57	71	59	63

Table S7. Regression line and r^2: Halyard Fluidshield 3 FFR fit for different testing categories. Grey regression line overlaid for each treatment method and category (y ~ mx + b) in Fig 2.

Treatment	Bending over	Deep breathing	Head side to side	Head up & down	Normal breathing (A)	Normal breathing (B)	Talking
Boil	$y = 117–25x$<br>$r^2 = 0.52$	$y = 110–26.2x$<br>$r^2= 0.30$	$y = 110–22.5x$<br>$r^2 = 0.38$	$y = 161–43.3x$<br>$r^2 = 0.82$	$y = 131–26.2x$<br>$r^2 = 0.17$	$y = 182–39.8x$<br>$r^2 = 0.37$	$y = 161–38.7x$<br>$r^2 = 0.36$
Cuckoo Rice cooker	$y = 212–16.8x$<br>$r^2 = 0.24$	$y = 186+0.6x$<br>$r^2 = 0.001$	$y = 174+4.9x$<br>$r^2 = 0.05$	$y = 203–3.2x$<br>$r^2 = 0.06$	$y = 198–6.13x$<br>$r^2 = 0.06$	$y = 196–4.7x$<br>$r^2 = 0.04$	$y = 198–1.03x$<br>$r^2 = 0.01$
Sous vide	$y = 212–27.9x$<br>$r^2 = 0.34$	$y = 218–21.9x$<br>$r^2= 0.28$	$y = 224–28.3x$<br>$r^2 = 0.38$	$y = 189–15.7x$<br>$r^2 = 0.02$	$y = 224–22.9x$<br>$r^2 = 0.34$	$y = 209–19.6x$<br>$r^2 = 0.19$	$y = 236–27.3x$<br>$r^2 = 0.41$

Table S8. Analysis of variance among treatment category groups and control Halyard Fluidshield 3 FFRs. Results of a 2-Way nested ANOVA comparing fit factor in seven test conditions before and after up to five rounds of heat treatment.

Treatment	Category	Rounds of treatment	p-value	F statistics
Boil	Bending over	1	0.00	31.93
		2	0.00	332.30
		3	0.00	234.99
	Deep breathing	1	0.02	16.68
		2	0.00	36.90
		3	0.00	111.65
	Head side to side	1	0.01	20.04
		2	0.00	635.47
		3	0.00	153.94
	Head up & down	1	0.00	32.98
		2	0.00	169.38
		3	0.00	369.88
	Normal breathing (A)	1	0.04	8.82
		2	0.07	6.22
		3	0.00	342.68
	Normal breathing (B)	1	0.04	8.88
		2	0.12	3.98
		3	0.00	102.08
	Talking	1	0.22	2.17
		2	0.21	2.19
		3	0.01	21.18
Cuckoo Rice cooker	Bending over	1	0.37	1.00
		2	0.37	1.00
		3	0.24	1.89
		4	0.09	5.09
		5	0.12	3.85
	Deep breathing	1	0.37	1.00
		2	0.37	1.00
		3	0.37	1.00
		4	0.37	1.00
		5	0.37	1.00
	Head side to side	1	0.37	1.00
		2	0.37	1.00
		3	0.37	1.00
		4	0.37	1.00
		5	0.37	1.00
	Head up & down	1	0.37	1.00
		2	0.37	1.00
		3	0.37	1.00
		4	0.37	1.00
		5	0.37	1.00
	Normal breathing (A)	1	0.37	1.00
		2	0.37	1.00
		3	0.37	1.00
		4	0.37	1.00

		5	0.37	1.00
	Normal breathing (B)	1	0.37	1.00
		2	0.37	1.00
		3	0.37	1.00
		4	0.19	2.50
		5	0.37	1.00
	Talking	1	0.37	1.00
		2	0.72	0.15
		3	0.37	1.00
		4	0.37	1.00
		5	0.65	0.24
Sous vide	Bending over	1	0.24	1.90
		2	0.16	3.02
		3	0.37	1.00
		4	0.04	9.05
		5	0.00	262.46
	Deep breathing	1	0.37	1.00
		2	0.37	1.00
		3	0.37	1.00
		4	0.12	3.99
		5	0.01	19.00
	Head side to side	1	0.37	1.00
		2	0.37	1.00
		3	0.37	1.00
		4	0.12	4.00
		5	0.00	307.45
	Head up & down	1	0.37	1.00
		2	0.37	1.00
		3	0.37	1.00
		4	0.12	3.92
		5	0.00	34.84
	Normal breathing (A)	1	0.37	1.00
		2	0.32	1.00
		3	0.37	1.00
		4	0.13	3.59
		5	0.01	31.12
	Normal breathing (B)	1	0.37	1.00
		2	0.37	1.00
		3	0.37	1.00
		4	0.12	3.87
		5	0.13	3.67
	Talking	1	0.37	1.00
		2	0.76	0.11
		3	0.37	1.00
		4	0.49	0.57
		5	0.04	8.76

Table S9. Filtration efficiency for control and treated Halyard Fluidshield 3 FFRs. None of the treatment methods tested reduces Halyard Fluidshield 3 FFR filtration performance below 95%. The pressure drop across N95 FFR was determined at a flow rate of 10 lpm.

Treatment	Rounds of treatment	Filtration Efficiency (FE)			Pressure Drop	
		Particle Size (μm)	F.E. of FFR Area 1 (%)	F.E. of FFR Area 2 (%)	FFR Area 1 (mmH2O)	FFR Area 2 (mmH2O)
Control	0	0.3	99.76	98.09	38.1	38.1
		0.5	99.88	98.68		
		1	99.94	99.35		
		2.5	99.98	99.85		
		5	99.89	99.89		
		10	99.88	100.00		
Boil	1	0.3	99.95	99.77	33.0	30.5
		0.5	100.00	99.78		
		1	100.00	100.00		
		2.5	100.00	100.00		
		5	100.00	100.00		
		10	100.00	100.00		
	2	0.3	100.00	99.64	38.1	30.5
		0.5	100.00	99.80		
		1	100.00	99.89		
		2.5	100.00	99.92		
		5	100.00	99.61		
		10	100.00	99.64		
	3	0.3	98.31	98.85	35.6	27.9
		0.5	98.76	98.61		
		1	99.45	98.98		
		2.5	99.65	100.00		
		5	99.38	100.00		
		10	99.42	100.00		
Cuckoo Rice Cooker	1	0.3	100.00	99.79	27.9	27.9
		0.5	100.00	99.75		
		1	100.00	99.80		
		2.5	100.00	100.00		
		5	100.00	100.00		
		10	100.00	100.00		
	2	0.3	100.00	98.89	33.0	30.5
		0.5	100.00	98.90		
		1	100.00	98.83		
		2.5	100.00	99.08		
		5	100.00	100.00		
		10	100.00	100.00		
	3	0.3	100.00	99.77	35.6	30.5
		0.5	100.00	99.89		
		1	100.00	99.96		
		2.5	100.00	99.92		
		5	100.00	99.72		
		10	100.00	97.44		
	4	0.3	99.52	97.16	30.5	33.0
		0.5	99.68	96.56		

		1	99.70	97.82		
		2.5	99.85	98.61		
		5	100.00	99.10		
		10	100.00	99.34		
	5	0.3	97.21	96.55	25.4	27.9
		0.5	98.79	99.36		
		1	97.34	99.39		
		2.5	96.33	100.00		
		5	100.00	100.00		
		10	100.00	100.00		
Sous vide	1	0.3	99.90	97.73	38.1	30.5
		0.5	99.98	98.73		
		1	99.90	97.12		
		2.5	100.00	97.57		
		5	100.00	100.00		
		10	100.00	100.00		
	2	0.3	95.43	98.48	27.9	27.9
		0.5	97.37	99.84		
		1	97.93	99.82		
		2.5	98.89	98.91		
		5	99.46	100.00		
		10	99.39	100.00		
	3	0.3	99.49	98.33	27.9	27.9
		0.5	99.67	99.88		
		1	99.86	99.91		
		2.5	99.98	99.90		
		5	99.93	99.52		
		10	99.78	100.00		
	4	0.3	96.61	96.92	25.4	25.4
		0.5	97.71	97.62		
		1	97.78	96.85		
		2.5	97.30	97.22		
		5	100.00	99.21		
		10	100.00	100.00		
	5	0.3	100.00	98.79	30.5	27.9
		0.5	100.00	99.28		
		1	100.00	99.69		
		2.5	100.00	99.81		
		5	100.00	99.93		
		10	100.00	99.80		

Table S10. Pressure drop across the N95 material for control and punctured Halyard Fluidshield 3 FFRs. The pressure drop across the N95 FFR material was determined at a flow rate of 10 lpm before and after one, two, and three punctures made with an 18-gauge needle.

Number of pinholes	Pressure Drop (mmH_2O)	
	Across FFR Area 1	Across FFR Area 2
0	30.5	27.9
1	12.7	15.2
2	7.6	5.1
3	2.5	2.5

References

1. Daeschler, S. C. *et al.* Effect of moist heat reprocessing of N95 respirators on SARS-CoV-2 inactivation and respirator function. *Cmaj* **192**, E1189–E1197 (2020).

2. Kumar, A. *et al.* N95 Mask Decontamination using Standard Hospital Sterilization Technologies. *undefined* doi:10.1101/2020.04.05.20049346.

3. Fischer, R. *et al.* Assessment of N95 respirator decontamination and re-use for SARS-CoV-2. (2020).

4. Rockey, N. *et al.* Humidity and Deposition Solution Play a Critical Role in Virus Inactivation by Heat Treatment of N95 Respirators. *Msphere* **5**, (2020).

5. Massey, T. *et al.* Quantitative form and fit of N95 filtering facepiece respirators are retained and coronavirus surrogate is inactivated after heat treatments. *undefined* doi:10.1101/2020.04.15.20065755.

6. Lore, M. B., Heimbuch, B. K., Brown, T. L., Wander, J. D. & Hinrichs, S. H. Effectiveness of Three Decontamination Treatments against Influenza Virus Applied to Filtering Facepiece Respirators. *Ann Occup Hyg* **56**, 92–101 (2012).
